# Supplementary material for: An integrated multi-criteria decision-making model for long-term planning of UAVs in disaster management
Source: PLoS One. 2026 Jan 6;21(1):e0340303. doi: 10.1371/journal.pone.0340303 (PMC12774348; doi:10.1371/journal.pone.0340303)
Supplement: Attachment — Submitted filename: 00 ResponseTo Reviewers.docx [file pone.0340303.s002.docx]

**Manuscript Number and Title:** PONE-D-25-47729, An Integrated Multi-Criteria Decision-Making Model for Long-Term Planning of UAVs in Disaster Management.

**Response to Reviewer Comments**

| **Reviewer 1** | |
| --- | --- |
| **Reviewer Suggestion/Comment** | **Author Action/Revision** |
| Overall assessment of the manuscript especially regarding the framework, identified gap, generalizability of the methodology, clarity and reproducibility. | We are grateful for the reviewer’s feedback and appreciate the insightful and constructive comments provided. |
| **1.** While the integration is well-executed, the individual components (AHP, GP) are well-established. However, this is an incremental rather than a groundbreaking theoretical advances.  The choice of the four specific goals (number of bases, distance, unairworthy days, cost) is presented without a strong theoretical or literature-based justification for why this specific set is the most critical. A stronger theoretical paper would defend this choice more rigorously. | We have revised Section 3.1 p.8 and justify why we use the designated goals (number of bases, distance, unairworthy days, cost) in the study supporting the choices with also some studies in literature. |
| **2.** I have a big doubt on how the study’s model is practically applicable in the area where topography is very crucial. Please provide how the model’s specific assumptions are made with the practical consideration. Kindly address the following critical observations.   1. Line-of-Sight (LoS) and Signal Reliability: UAVs, especially for BVLOS (Beyond Visual Line of Sight) operations, often rely on radio or satellite communication. Mountains, hills, and urban canyons can block these signals, making a location that is geographically "close" in terms of distance completely unusable. The paper's model only considers a flat, 2D distance (R_ij), which is a massive oversimplification. 2. Take-off and Landing Requirements: A candidate base must have a sufficiently large, flat, and clear area for safe take-off and landing. A location that is optimal on a map might be on a steep slope, in a dense forest, or in an urban area with no clear space. 3. Wind Patterns and Microclimates: Topography dramatically influences local weather. A valley might be prone to fog, a mountain pass to high winds, and a coastal area to salt spray. The paper's single metric of "unairworthy days" (UAD_i) is too coarse; it doesn't capture how topography creates localized, persistent hazardous conditions that would make a specific site unsuitable, regardless of the regional weather data. 4. Flight Path Obstacles: The optimal path from a base to a Disaster Activity Zone (DAZ) is not a straight line. It must navigate around terrain features. The paper's "flight distance" is a simple straight-line radius, ignoring that the actual flight path might be much longer and more energy-consuming to go around a mountain range. 5. Is it around 5 km radius of coverage in your model??? | In this study, we concentrate on the long-term planning of UAV operations, with particular emphasis on the selection and designation of appropriate bases. We assume that there is a sufficient number of well-established bases within the area of interest from which an appropriate subset can be selected for the UAVs under consideration, consistent with typical facility location problems. We also assume that the UAVs in this study operate at high altitudes, use mainly satellite communication, have substantial observational capacity with high-tech imaging systems and are capable of extended flight durations. In this respect:   1. Line-of-Sight (LoS) and Signal Reliability: The UAVs under consideration operate at high altitudes and use mainly satellite communication. 2. Take-off and Landing Requirements: In facility location problems a selection is made among a set of existing facilities. In our case, a selection of air bases is made among existing well-established air bases, which normally meet take-off and landing requirements of UAVs. 3. Wind Patterns and Microclimates: The weather data used is specific to the region where an existing air base is located and indicates the expected number of unairworthy days. While such data may present limitations for short-term planning, they are suitable for long-term planning purposes. 4. Flight Path Obstacles: The UAVs examined in this study operate at high altitudes, are equipped with advanced imaging systems that provide substantial observational capability and can sustain long flight durations. 5. The radius of coverage in the model: The UAVs examined in this study do not have a predefined coverage range, owing to their ability to operate at high altitudes and their extensive observational capability enabled by high-resolution, advanced camera systems.   Accordingly, we have revised Section 3.1 p.8 and incorporated a new paragraph-the final paragraph of the subsection-clarifying the specific operational assumptions for the reader, as suggested by the reviewer.  Additionally, as it would constitute a valuable contribution to literature, we propose in the Conclusion and Future Work section that a study emphasizing short-term planning—accounting for daily weather conditions, topography, route patterns, and time scheduling aspects—should be pursued as a new avenue for future research. |
| **3.** In several key places, the paper uses a comma (,) as a decimal separator for the objective function value, which is a percentage.  Critical Examples:  Optimal Objective Function Value ... = 7,2% (Page 21, Table 3)  ... = 7,8% (Page 24, Table 5)  ... = 14,4% and ... = 15,6% (Page 26 & 28, Tables 7 & 9)  Number formats for km and dollars are confusing.  Page 16…..total unairworthy days, and total cost turn out to be 8, 4.792.590, 833, and 12.580.890  Page 16 …… Total Flight Distance (km) in table 4….4.046.475 ……….. 4.792.590  Page 16…….. Total Cost (dollars) in table 4…… 10.582.690…… 12.580.890….. 1.998.197  Check the entire manuscript or it is a coding? | We have revised the whole manuscript and use a dot (.) as a decimal separator in line with the international numeric standard, as suggested by the reviewer.  To avoid confusion, we also delete the thousand separators. |
| **Reviewer 2** | |
| **Reviewer Suggestion/Comment** | **Author Action/Revision** |
| Overall assessment of the manuscript. | We are thankful for the reviewer’s insightful and constructive feedback. |
| **1.**Empirical Data Validation: The study relies on simulated (“generic”) data. While this is acceptable for methodological development, the absence of real-world or semi-real data limits external validity.  Suggestion: Incorporate or reference a case study using actual disaster-region data (e.g., UAV deployment during forest fires, floods, or earthquakes in Turkey) to demonstrate the model’s practical applicability. | We have revised Section 4, Application Results and Discussion, and separated it into two subsections.   - Subsection 4.1 covers our illustrative example. - The newly added subsection 4.2. covers a real-world scenario, the case of an earthquake in Türkiye as suggested by the reviewer.   Abstract and Conclusion parts have been updated to cover the newly added scenario.  The code and data associated with the application have been updated on GitHub to cover also the newly added scenario. (https://github.com/fkasimoglu/Integrated-MCDM-Model-for-UAVs) |
| 2. Figures 3–6 are informative but could be enhanced with color or clearer legends to help distinguish bases and disaster activity zones visually. | Figures 1,3,4,5 and 6 are visually enhanced to help distinguish bases and disaster activity zones, as suggested by the reviewer. |
| 3. Consider including a comparative chart showing percentage improvements across all cases (1–3) to make the improvements easier to interpret. | Figure 7 is added to make the improvements easier to interpret, as suggested by the reviewer. |
