# Supplementary material for: An integrated multi-criteria decision-making model for long-term planning of UAVs in disaster management
Source: PLoS One. 2026 Jan 6;21(1):e0340303. doi: 10.1371/journal.pone.0340303 (PMC12774348; doi:10.1371/journal.pone.0340303)
Supplement: Attachment — Submitted filename: review_report_PLOS_ONE_r1.docx [file pone.0340303.s003.docx]

Comments:

Thank you for submitting the revised version of your manuscript, "An Integrated Multi-Criteria Decision-Making Model for Long-Term Planning of UAVs in Disaster Management." I have carefully reviewed the changes made in response to the previous round of comments.

The authors have successfully addressed the key concerns raised in the first review. In particular, section 3.1 p.8. has been revised broadly to capture key comments raised by incorporating operational assumptions that can benefit the readers. Accordingly, conclusion of the research show clear future scope based on whatever the theoretical contribution that the paper demonstrates. In addition, comment 3 has been taken care throughout the manuscript.

Thank you and best wishes!
